# Multi-center, single-blind randomized controlled trial comparing functional electrical stimulation therapy to conventional therapy in incomplete tetraplegia

**DOI:** 10.3389/fresc.2022.995244

**Published:** 2022-09-09

**Authors:** Kim D. Anderson, Radha Korupolu, Kristin E. Musselman, Jacqueline Pierce, James R. Wilson, Nuray Yozbatiran, Naaz Desai, Milos R. Popovic, Lehana Thabane

**Affiliations:** ^1^ MetroHealth Rehabilitation Institute, MetroHealth System, Cleveland, OH, United States; ^2^ Department of Physical Medicine and Rehabilitation, Case Western Reserve University School of Medicine, Cleveland, OH, United States; ^3^ Department of Physical Medicine and Rehabilitation, McGovern Medical School, The University of Texas Health Science Center at Houston, Houston, TX, United States; ^4^ The Institute of Rehabilitation / Research (TIRR) Memorial Hermann, Houston, TX, United States; ^5^ The KITE Research Institute, University Health Network, Toronto, ON, Canada; ^6^ Department of Physical Therapy, University of Toronto, Toronto, ON, Canada; ^7^ HealthTech Connex Centre for Neurology Studies/Neuromotion Physiotherapy, Vancouver, BC, Canada; ^8^ Krembil Research Institute-University Health Network, Toronto, ON, Canada; ^9^ Institute of Biomedical Engineering, University of Toronto, Toronto, ON, Canada; ^10^ CRANIA, University Health Network, Toronto, ON, Canada; ^11^ Department of Health Research Methods, Evidence, and Impact, Faculty of Health Sciences, McMaster University, Hamilton ON, Canada; ^12^ Biostatistics Unit, St. Joseph’s Healthcare, Hamilton, ON, Canada; ^13^ Faculty of Health Sciences, University of Johannesburg, Johannesburg, Gauteng, South Africa

**Keywords:** functional electrical simulation (FES), spinal cord injury, tetraplegia, therapy, rehabilation

## Abstract

**Background:**

Loss of upper extremity function after tetraplegia results in significant disability. Emerging evidence from pilot studies suggests that functional electrical stimulation (FES) therapy may enhance recovery of upper extremity function after tetraplegia. The aim of this trial was to determine the effectiveness of FES therapy delivered by the Myndmove stimulator in people with tetraplegia.

**Methods:**

A multi-center**,** single-blind, parallel-group, two-arm, randomized controlled trial was conducted comparing FES to conventional therapy in adults (≥18 years) with C4–C7 traumatic incomplete tetraplegia between 4 and 96 months post-injury, and with a baseline spinal cord injury independence measure III -self-care (SCIM III-SC) score of ≤10. Participants were enrolled at four SCI-specialized neurorehabilitation centers in the U.S. and Canada. Participants were stratified by center and randomized in a 1:1 ratio to receive either 40 sessions of FES or conventional therapy targeting upper extremities over a 14-week period. Blinded assessors measured SCIM III, Toronto Rehabilitation Institute Hand Function Test, and Graded Redefined Assessment of Strength, Sensibility, and Prehension at baseline, after 20th session, after 40th session or 14 weeks after 1st session, and at 24 weeks after 1st session. The primary outcome measure was change in SCIM III-SC from baseline to end of the treatment. Based on the primary outcome measure, a sample size of 60 was calculated. Seventeen participants' progress in the study was interrupted due to the COVID-19 lockdown. The protocol was modified for these participants to allow them to complete the study.

**Results:**

Between June 2019 to August 2021, 51 participants were randomized to FES (*n* = 27) and conventional therapy (*n* = 24). Both groups gained a mean of 2 points in SCIM-SC scores at the end of treatment, which was a clinically meaningful change. However, there was no statistically significant difference between the groups on any outcomes.

**Conclusion:**

Forty sessions of FES therapy delivered by the MyndMove stimulator are as effective as conventional therapy in producing meaningful functional improvements that persist after therapy is completed. Limitations of this study include the impact of COVID-19 limiting the ability to recruit the target sample size and per-protocol execution of the study in one-third of the participants.

**Registration:**

This trial is registered at www.ClinicalTrials.gov, NCT03439319.

## Introduction

Recovery of upper extremity function after spinal cord injury (SCI) is reported as the highest priority by individuals living with tetraplegia, which is essential to improve independence and quality of life after SCI (1). Therefore, it is vital to study and develop interventions to improve the upper extremity motor deficits following SCI. Conventional rehabilitation is the most common approach to restoring motor function after SCI. However, therapeutic interventions beyond conventional therapy are limited in availability. Hence, there is an interest in combining rehabilitation therapy with various interventions including functional electrical stimulation (FES) to enhance the effects of conventional rehabilitation therapy.

Emerging evidence suggests that FES therapy, when combined with conventional rehabilitation interventions, results in greater improvement in motor function after SCI and stroke (2–4). FES provides small electrical pulses to stimulate motor neurons *via* surface or implanted electrodes to facilitate muscle contraction during a functional activity (5, 6). There are different approaches to using FES therapy to treat individuals living with SCI. One of the techniques is the short-term therapeutic application of FES to improve voluntary hand function. During this type of FES therapy, individuals are asked to perform or attempt functional movements with their paralyzed extremity, combined with electrical stimulation of muscles responsible for producing that movement. This process is repeated and practiced with multiple functional movements during a session. Many FES systems have been developed for this purpose, including the NESS H200, the Bionic Glove and its newer version the HandEstim Wireless Hand Stimulator, the Compex Motion system and its later commercial version the MyndMove stimulator (5–12).

FES therapy delivered by the Compex Motion system was studied in a single-site pilot randomized controlled trial (RCT). The efficacy of 40 h of FES therapy (Compex Motion system) with conventional therapy (*n* = 9) was compared to conventional therapy alone (*n* = 12) to improve grasp function in people with subacute traumatic incomplete cervical SCI (13). The key outcomes were changes in Functional Independence Measure (FIM) self-care subscores, Spinal Cord Independence Measure III (SCIM) self-care subscores, and Toronto Rehabilitation Institute Hand Function Test (TRI-HFT) performed at baseline and after the intervention at the end of 8 weeks. The FES therapy group had greater improvement in SCIM self-care and TRI-HFT scores. These findings were consistent with previous studies of FES therapy performed by this group in individuals with stroke (14).

Based on these single-center clinical studies of FES therapy delivered by the Compex Motion system, the commercial version of the stimulator (named MyndMove) was developed for use as a neurorehabilitation intervention tool applicable across a broad range of inpatient and outpatient clinical settings. The MyndMove stimulator is cleared as a non-significant risk device by the U.S. Food and Drug Administration and exempt from an Investigational Device Exemption (IDE; reference file Q131135). The MyndMove stimulator is used to deliver non-invasive FES therapy *via* surface electrodes to pursue target muscle contractions to facilitate functional movements. It is hypothesized that the massed practice of these FES-facilitated functional movements induces neuroplasticity, restoring the voluntary function of the upper extremities.

As a next step, a large multi-center RCT was conducted to compare the effectiveness of FES therapy (delivered by the MyndMove stimulator) to conventional therapy (C.T.) in improving upper extremity motor function in individuals with incomplete cervical SCI. We hypothesized that 40 h of FES therapy would result in greater improvements in upper extremity function and quality of life in individuals with cervical traumatic incomplete subacute to early chronic SCI compared to an equal dose of conventional therapy.

## Materials and methods

### Summary of design and participants

This multi-site study was designed as a parallel-group, two-arm, single-blind, RCT comparing FES therapy to C.T. in individuals with incomplete tetraplegia. The detailed protocol was previously published (12). A summary description is provided below.

**Figure 1:**
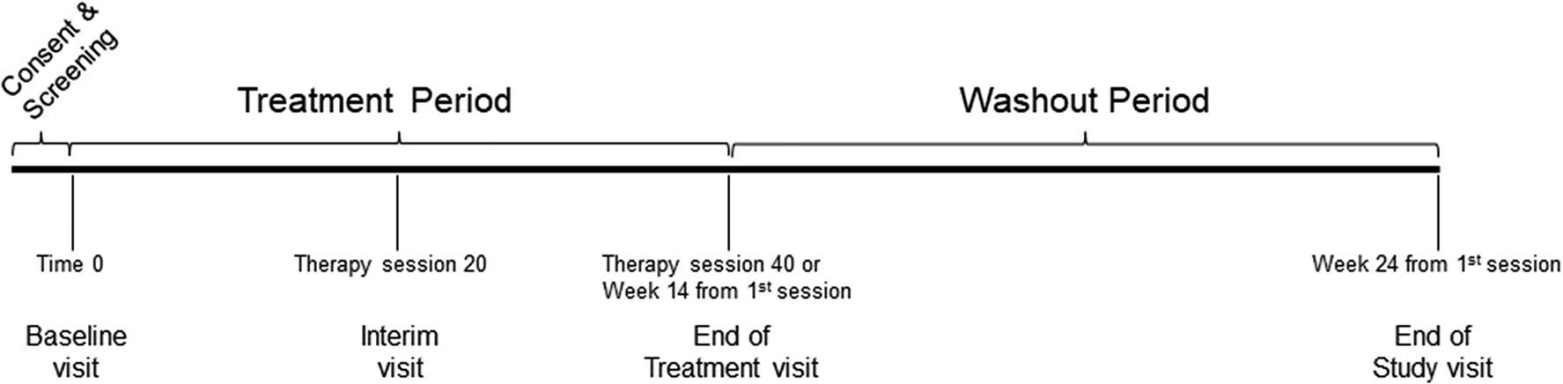
Study design and timeline. The baseline visit was considered Time 0 and randomization occured within three days after the visit. The treatment period included forty therapy sessions that had to be completed in a time window of no more than fourteen weeks from the first therapy session. The washout period was from the last therapy until twenty-four weeks from the first therapy session.

Eligible participants were individuals age 18 and older, with C4–7 traumatic SCI, ASIA Impairment Scale (AIS) grades B-D, between 4 and 96 months post-injury, and with a baseline SCIM III self-care (SCIM-SC) subscale score ≤10 (full inclusion and exclusion criteria published in Anderson et al. (12). Participants were enrolled at four SCI-specialized neurorehabilitation centers in the U.S. and Canada. A sample size of 60 was calculated based on the hypothesis that the mean difference in SCIM-SC in the FES group is better than the C.T. group after 14 weeks of treatment [full description of sample size calculation described in Anderson et al. (12) as well as rationale for selecting the SCIM-SC as the primary outcome]. Figure 1 depicts the study design and timeline.

### Allocation and intervention

Participants were stratified by study center and randomized in a 1:1 ratio based on permutated blocks of random sizes to one of the two treatment arms below:

1. FES therapy (intervention group)—participants engaged in 36–40 1-hour sessions of FES therapy within a 14-week period.
2. C.T. (active control group)—participants engaged in 36–40 1-hour sessions of upper limb conventional therapy within a 14-week period.

FES therapy was provided using the MyndMove device (MyndTec, Inc., Mississauga, Canada) (12). Based on each participant's clinical presentation and goals, therapists could choose from eight movement patterns during each 1-hour therapy session. The movement patterns were palmar grasp, lateral pinch grasp, pinch grasp, lumbrical grasp, tripod grasp, side reach with finger extension, forward reach and grasp, and hand to mouth [see Anderson et al. (12) for full description]. These preprogrammed movement patterns are intended to facilitate task-specific movements that are practiced in a massed or distributed fashion. The FES therapy group did not receive C.T.

Conventional therapy (C.T.) served as an active control and was intended as a time equivalent to the FES group. Also based on each participant's clinical presentation and goals, therapists could choose from seven categories of conventional upper limb rehabilitation therapy [see Anderson et al. (12) for full description]. The categories were facilitation of reach or prehension movements, bilateral task-specific movements, range of motion and mobilization of joints, splinting, sensorimotor stimulation, electrical stimulation (single muscles for strength, not function), and reduction of edema. The type, frequency, and duration of each category was not prescribed aside from fitting within the 1-hour therapy session time frame to meet the 36–40 sessions within a 14-week period.

For both groups, therapy was delivered in 1-hour sessions with participants engaging in 3–5 sessions each week. Completing 36–40 sessions within a 14-week period was considered a successful completion of the intervention protocol.

### Assessments and analyses

Therapists not involved with treatment performed blinded assessments of three functional outcome measures [SCIM III, TRI-HFT, Graded Redefined Assessment of Strength, Sensibility, and Prehension (GRASSP)] at Baseline, Interim (after 20th session), End of Treatment (after 40th session/14 weeks from 1st session), and End of Study (24 weeks from 1st session). The primary outcome (the change in SCIM-SC subscale score from Baseline to End of Treatment) was derived from the full SCIM III. Secondary analyses of all time points compared to baseline included SCIM-SC and mobility subscores, the TRI-HFT subscores, and the GRASSP subscores.

The SCIM-SC was chosen as the primary outcome based on the results of the pilot study (15) and because it is recommended as the “Supplemental Highly Recommended” outcome measure to use when assessing function in persons with SCI (SCI Common Data Elements). The SCIM, TRI-HFT, and GRASSP were all designed specifically for SCI (Catz et al., 1997 (16); Kapadia et al., 2012 (17); Kalsi-Ryan et al., 2009 (18), and have all been validated in SCI (Itzkovich et al., 2007 (19); Kapadia et al., 2012 (17); Kalsi-Ryan et al., 2012 (20). All components of each outcome measure were used.

Participant demographics and disease characteristics were captured at baseline. Unblinded assessments were obtained on 9 of the 22 Spinal Cord Injury-Quality of Life (SCI-QOL) outcome scales at Baseline, End of Treatment, and End of Study. All adverse events were recorded, graded, and evaluated for relationship to study intervention.

Primary, secondary, and sensitivity analyses were described in detail in the protocol publication (12). The statistician was blind to study group. Briefly, we adopted an intention-to-treat principle to analyze all outcomes and used multiple imputation to handle missing data. We used analysis of covariance adjustiging for baseline score for all outcomes. The criterion for statistical significance was set at alpha = 0.05. The results are reported as estimate of effect, corresponding 95% confidence interval (CI), and associated *p*-value. All *p*-values are reported to three decimal places with those less than 0.001 reported as *p* < 0.001. We performed some sensitivity analyses to assess to robustness of the results on the primary outcome of SCIM-SC subscale score using: (i) per-protocol approach that included only those participants that adhered to the protocol; (ii) last observation carried forward to handle missing data; (iii) adjusting for age, SCIM-SC baseline subscale score. All analyses were performed using SAS 9.4 (Cary, NC).

### Unplanned interruption due to COVID-19

Seventeen participants (Supplementary Table S1) whose therapy sessions were paused at the time of the COVID-19 lockdown (March 2020) in the U.S. and Canada were dealt with in the following manner when each resumed activity in the trial:

1. Those who completed less than or equal to 20 sessions at the time of the lockdown (*N* = 7 of 17) were asked to restart the study with a new baseline assessment of SCIM, TRI-HFT, and GRASSP,
2. Those who completed more than 20 sessions (*N* = 3 of 17 plus 1 passed away prior to restart) were restarted at the therapy session number they had paused at and completed the remaining sessions (a 2nd interim assessment of SCIM, TRI-HFT, and GRASSP was performed prior to restarting the study in these participants), and
3. Participants who completed 40 sessions at the time of the lockdown but missed their follow-up assessments (*N* = 6 of 17) had an end of treatment assessment performed when studies were restarted in their respective institutions.

### Ethics approval

This study had ethics approval from: MetroHealth System Institutional Review Board (IRB18-0751); University Health Network Research Ethics Board (REB17-6029); University of Texas Health Science Center IRB (HSC-MS-18–0862); Advarra IRB for HealthTech Connex Centre for Neurology Studies (Pro00030094); as well as approval from the US Army Medical Research and Materiel Command, Office of Research Protections and Human Research Protection Office.

## Results

### Participants

The first participant was enrolled in June 2019 and the final participant was enrolled in August 2021. Table 1 shows the CONSORT flow diagram for the study. Of the 69 individuals assessed, 51 were randomized to a study group. The COVID-19 pandemic did impact enrollment. As a result, the randomization target of 60 was not met.

**Table 1:** CONSORT flow diagram.

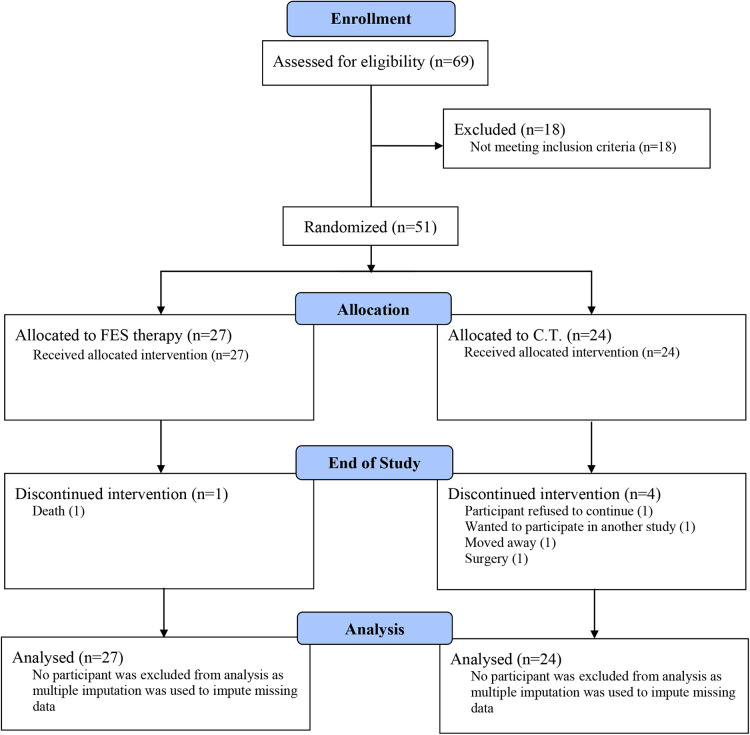

Table 2 contains site distribution, demographic, and injury characteristic data for the 51 randomized participants. Based on these specific parameters, there were no significant differences between the FES group compared to the C.T. group, but there was a trend for individuals in the FES group to have a higher level of injury and to be slightly longer post-injury than those in the C.T. group.

**Table 2:** Demographics and social status.

Variable	FES therapy (*n* = 27)	Conventional therapy (*n* = 24)
Study Site [*n* (%)]		
Cleveland	7 (29.17)	7 (29.17)
Toronto	4 (14.81)	5 (20.83)
Houston	9 (33.33)	6 (25.00)
Vancouver	7 (29.17)	6 (25.00)
ASIA Impairment Scale [*n* (%)]		
B	10 (37.0)	9 (37.5)
C	11 (40.7)	10 (41.7)
D	6 (22.2)	5 (20.8)
Neurological Level of Injury [*n* (%)]		
C4	18 (66.7)	10 (41.7)
C5	5 (18.5)	7 (29.2)
C6	4 (14.8)	6 (25.0)
C7	0 (0)	1 (4.2)
Time post-injury (30-day months) [median (Q1, Q3)]	23.7 (12.9, 36.6)	17.6 (7.4, 27.8)
Sex (Male) [*n* (%)]	23 (85.19)	17 (70.83)
Age (y) [*n*, mean (SD)]	27, 40.00 (17.98)	24, 46.71 (17.25)
Race [*n* (%)]		
Caucasian or White	14 (51.85)	10 (41.67)
American Indian or Alaska Native or Aboriginal	0 (0)	0 (0)
Asian	4 (14.81)	2 (8.33)
Black or African American	4 (14.81)	2 (8.33)
Native Hawaiian or Other Pacific Islander White	0 (0)	0 (0)
Unknown	0 (0)	1 (4.17)
Other	0 (0)	1 (4.17)
Canadian participant	5 (18.5)	8 (33.3)
Ethnicity [*n* (%)]		
Caucasian	16 (59.26)	14 (58.33)
Aboriginal	0 (0)	0 (0)
Chinese	2 (7.41)	0 (0)
South Asian	0 (0)	3 (12.5)
Black	4 (14.8)	2 (8.33)
Filipino	0 (0)	0 (0)
Hispanic or Latino	2 (7.41)	3 (12.5)
Southeast Asian	0 (0)	1 (4.17)
Arab	0 (0)	1 (4.17)
West Asian, Korean or Japanese	1 (3.70)	0 (0)
Unknown	0 (0)	0 (0)
Other	2 (7.41)	0 (0)
Marital Status [*n* (%)]		
Never married	15 (55.56)	7 (29.17)
Married	7 (25.93)	8 (33.33)
Domestic partnership	1 (3.70)	1 (4.17)
Divorced	2 (7.41)	6 (25.00)
Separated	2 (7.41)	1 (4.17)
Widowed	0 (0)	1 (4.17)
Education (y) [*n*, mean (SD)]	27, 13.96 (2.08)	22, 14.50 (3.36)
Primary Occupation [*n* (%)]		
Paid work (employed/self-employed)	4 (14.81)	4 (16.67)
Homemaker	0 (0)	1 (4.17)
Student (including on the job training)	4 (14.81)	2 (8.33)
Retired (disability pension)	4 (14.81)	1 (4.17)
Retired (non-disability)	1 (3.70)	5 (20.83)
Unpaid work (volunteer)	0 (0)	0 (0)
Unemployed (none of the above)	13 (48.15)	10 (41.67)
Other	1 (3.70)	1 (4.17)
Family Income [*n* (%)]		
Under $15,000	3 (11.11)	4 (16.67)
$15,000 to $24,999	1 (3.70)	1 (4.17)
$25,000 to $34,999	0 (0)	0 (0)
$35,000 to $49,999	0 (0)	0 (0)
$50,000 to $74,999	2 (7.41)	0 (0)
$75,000 to $99,999	1 (3.70)	2 (8.33)
$100,000 and over	3 (11.11)	3 (12.50)
Refused to Answer	17 (62.96)	14 (58.33)
Pre-Injury Hand Preference [*n* (%)]		
Right hand	25 (92.59)	23 (95.83)
Left hand	2 (7.41)	1 (4.17)
Both hands	0 (0)	0 (0)
Post-Injury Hand Preference [*n* (%)]		
Right hand	13 (48.15)	13 (54.17)
Left hand	12 (44.44)	9 (37.50)
Both hands	2 (7.41)	2 (8.33)

N, number of participants; SD, standard deviation; Q1, first quartile; Q3, third quartile; Y, years.

Table 3 provides a summary of the frequency of categories utilized during the C.T. group therapy sessions and Table 4 provides a summary of the minutes spent in each category. The most frequent category used (and for the longest duration) during C.T. therapy sessions was facilitation of reach and prehension movements.

**Table 3:** Summary of total number of C.T. sessions for each C.T. category.

C.T. category	Mean	SD	Lower quartile	Median	Upper quartile	Minimum	Maximum
Facilitation of reach and prehension movements	30.42	8.39	28.00	33.00	36.50	10.00	39.00
Bilateral tasks training	19.17	11.97	9.50	22.50	28.00	0.00	39.00
Range of motion and mobilization of joints	23.08	15.26	3.50	28.50	37.50	0.00	40.00
Splinting	2.58	2.78	0.00	2.50	4.00	0.00	11.00
Sensorimotor stimulation (e.g., TENS, acupuncture, ..)	9.29	9.82	2.00	6.00	13.50	0.00	36.00
Electrical stimulation for focal muscle strengthening only	12.92	10.82	3.50	11.50	22.00	0.00	38.00
Reduction of Edema	0.54	1.72	0.00	0.00	0.00	0.00	8.00

C.T., conventional therapy; SD, standard deviation; TENS, transcutaneous electrical nerve stimulation.

**Table 4:** Summary of total time (minutes) spent in each C.T. category.

C.T. category	Mean	SD	Lower quartile	Median	Upper quartile	Minimum	Maximum
Facilitation of reach and prehension movements	772.00	412.99	509.00	654.00	1042.50	185.00	1840.00
Bilateral tasks training	336.71	220.66	145.00	383.00	470.00	0.00	830.00
Range of motion and mobilization of joints	333.48	303.24	50.00	318.00	475.00	0.00	1265.00
Splinting	43.67	67.54	0.00	20.00	52.50	0.00	245.00
Sensorimotor stimulation (e.g., TENS, acupuncture, ..)	273.17	464.74	35.00	70.00	237.50	0.00	2060.00
Electrical stimulation for focal muscle strengthening only	276.08	242.83	70.00	210.00	470.00	0.00	800.00
Reduction of Edema	5.83	17.73	0.00	0.00	0.00	0.00	80.00

C.T., conventional therapy; SD, standard deviation; TENS, transcutaneous electrical nerve stimulation.

### Primary outcomes

**Figure 2:**
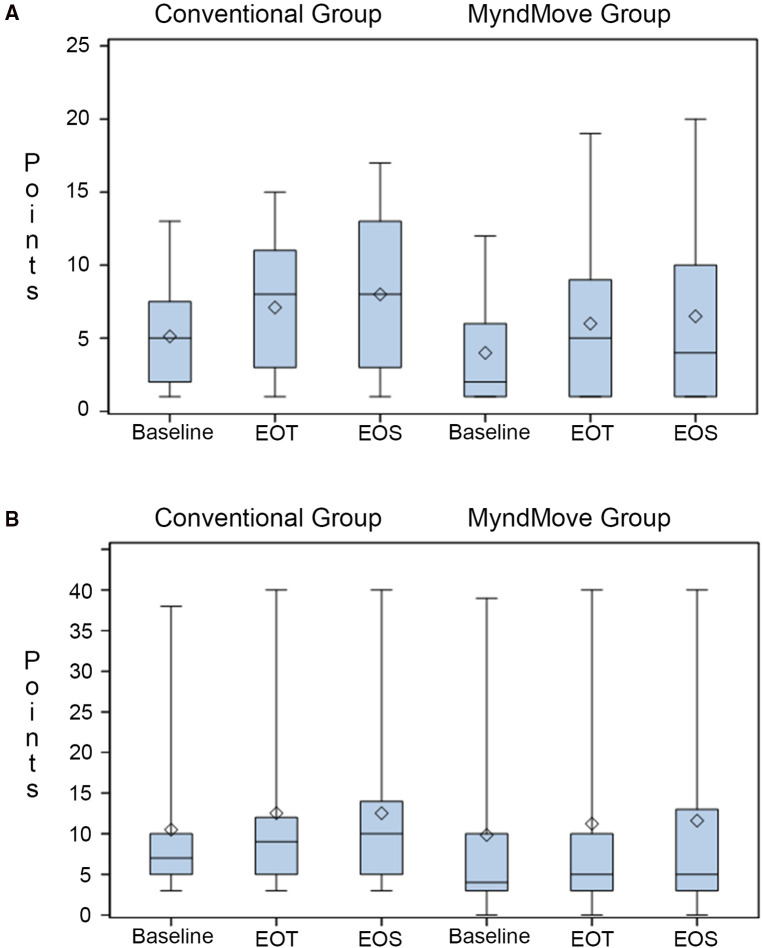
Box plots of spinal cord independence measure (SCIM) scale. (**A**) SCIM self-care subscale results. (**B**) SCIM mobility subscale results. Mean (line), median (diamond), quartile 1, and quartile 3 presented in each plot. EOT, end of treatment; EOS, end of study.

The primary outcome for the study is the change in SCIM-SC between baseline and end of treatment, hypothesizing that FES therapy is better than C.T. The SCIM-SC subscale ranges from 0 to 20 points. A change of 1.12 points is considered a small meaningful change and a change of 2.8 points is considered a substantial meaningful change (16). Figure 2A shows the mean, median, and upper and lower interquartile range for both groups. Both groups gained a mean of 2 points at the end of treatment, which persisted at the end of study (secondary outcome). However, there was no statistically significant difference between either group (Table 5).

**Table 5:** SCIM.

Variable^a^	Baseline		End of treatment		End of study		Outcome analysis	
	FES	CT	FES	CT	FES	CT	Effect estimate (95% CI)	*p*-value
Self Care subscale	4.00 (3.32) *N* = 27	5.13 (3.40) *N* = 24	6.00 (5.00) *N* = 26	7.11 (4.56) *N* = 19	6.50 (5.86) *N* = 26	8.00 (5.39) *N* = 19	−0.55 (−2.79, 1.69)	0.631
Mobility subscale	9.85 (11.75) *N* = 27	10.50 (9.26) *N* = 24	11.23 (12.47) *N* = 26	12.53 (11.42) *N* = 19	11.62 (13.01) *N* = 26	12.53 (10.89) *N* = 19	−0.60 (−2.60, 1.39)	0.552

SCIM, Spinal Cord Independence Measure; FES, functional electrical stimulation; CT, conventional therapy; CI, confidence interval; N, number of participants.

^a^ Data reported as: mean (SD), unless otherwise specified.

### Secondary outcomes

The SCIM mobility subscale includes various mobility items involving the upper and lower extremities and the subscore ranges from 0 to 40 points. The change in this subcore between groups across all time points was a secondary outcome. There is not a single meaningful change for the full mobility subscale (16), it is subdivided into the three room/toilet questions [small meaningful change = 0.58, substantial meaningful change = 1.45] and the six indoors/outdoors questions [small meaningful change = 0.78, substantial meaningful change = 1.95]. Figure 2B shows the mean, median, and upper and lower interquartile range for both groups. Both groups gained a mean of 2 points (across the full subscale) at the end of treatment, which persisted at the end of study. However, there was no statistically significant difference between groups (Table 5).

The GRASSP outcome data are presented as the change in total score of each subscale (Table 6) at end of treatment and end of study compared to baseline. The subscales are strength, sensibility, qualitative prehension, and quantitative prehension. The minimal detectable difference (MDD) and the minimal clinically important difference (MCID) are known for the GRASSP quantitative prehension subscale. The MDD is 9.7 points for the combined score and 6.0/5.3 for right/left side scores (17) and the MCID is 6.4 points (18). Though both groups gained points at the end of treatment that persisted at the end of study, the gains in the quantitative prehension subscore did not reach MDD or MCID and the differences were not statistically significant.

**Table 6:** GRASSP.

Subscale^a^	Baseline		End of treatment		End of study		Outcome analysis	
	FES	CT	FES	CT	FES	CT	Effect estimate (95% CI)	*p*-value
Strength total score	36.96 (20.23) *N* = 27	43.50 (15.04) *N* = 24	40.73 (22.44) *N* = 26	49.32 (14.56) *N* = 19	41.35 (22.73) *N* = 26	48.42 (15.90) *N* = 19	−1.00 (5.44,3.44)	0.658
Sensibility total score	23.15 (12.96) *N* = 27	28.88 (12.45) *N* = 24	24.88 (13.99) *N* = 26	29.58 (11.96) *N* = 19	23.77 (15.07) *N* = 26	29.32 (12.00) *N* = 19	−2.68 (9.85,4.49)	0.464
Qualitative prehension total score	7.85 (6.97) *N* = 27	9.54 (5.09) *N* = 24	8.85 (7.64) *N* = 26	10.16 (6.41) *N* = 19	9.31 (7.63) *N* = 26	11.42 (6.27) *N* = 19	−2.68 (9.85,4.49)	0.464
Quantitative prehension total score	16.56 (13.49) *N* = 27	24.04 (11.56) *N* = 24	19.81 (13.73) *N* = 26	26.42 (13.49) *N* = 19	20.81 (14.70) *N* = 26	27.42 (13.81) *N* = 19	−1.57 (4.90,1.77)	0.357

GRASSP, Graded Redefined Assessment of Strength, Sensibility, and Prehension; SD, standard deviation; FES, functional electrical stimulation; CT, conventional therapy; CI, confidence interval; N, number of participants.

^a^ Data reported as: mean (SD), unless otherwise specified.

The TRI-HFT outcome data are also presented as the change in total score of each subscale (Table 7) at end of treatment and end of study compared to baseline. The subscales are object manipulation, wooden block, cylinder torque, credit card force, wooden bar thumb displacement length, and wooden bar finger displacement length. The minimal detectable change (MDC) with 95% confidence intervals is 9.1 for the object manipulation subscale and 9.2 for the wooden block subscale (calculated using the formula MDC = 1.96 × SEM × √2 (19), by author ND using a subacute SCI dataset provided by author MP; SEM was calculated using the formula SEM = SD/√N as the ICC of test-retest reliability for the dataset used was not available). The MDC was achieved for the object manipulation subscale in both groups at the end of treatment (object manipulation = 10.14 points in FES group, 11.18 points in C.T. group), but these changes were not statistically significant. For the wooden block subscale, the FES group exceeded the MDC (12.81 points) at the end of treatment while the C.T. group did not (1.12 points).

**Table 7:** TRI-HFT.

Subscale^a^	Baseline		End of treatment		End of study		Outcome analysis	
	FES	CT	FES	CT	FES	CT	Effect estimate (95% CI)	*p*-value
Object manipulation score	53.59 (35.93) *N* = 27	75.71 (33.27) *N* = 24	63.73 (39.45) *N* = 26	86.89 (36.54) *N* = 19	65.00 (39.27) *N* = 26	87.47 (39.31) *N* = 19	−4.58 (13.64,4.48)	0.321
Wooden block score	48.96 (32.33) *N* = 27	78.67 (35.10) *N* = 24	61.77 (39.18) *N* = 26	79.79 (36.73) *N* = 19	65.88 (39.31) *N* = 26	86.78 (35.75) *N* = 18	7.96 (10.95,26.87)	0.408
Cylinder torque^b^	1.41 (2.57) *N* = 27	1.56 (2.84) *N* = 24	1.58 (2.65) *N* = 26	1.80 (2.79) *N* = 19	1.96 (2.95) *N* = 26	2.69 (4.16) *N* = 19	0.78 (−2.41,0.85)	0.344
Credit card force^b^	2.46 (4.36) *N* = 26	2.10 (2.59) *N* = 24	2.71 (4.47) *N* = 26	2.05 (2.73) *N* = 19	3.18 (5.18) *N* = 26	3.86 (6.53) *N* = 19	−1.03 (−3.68,1.63)	0.446
Wooden bar, thumb displacement length^b^	4.71 (13.03) *N* = 27	10.21 (18.00) *N* = 24	6.96 (14.85) *N* = 26	13.95 (16.29) *N* = 19	8.38 (15.73) *N* = 26	15.16 (19.96) *N* = 19	0.81 (−8.17,9.79)	0.858
Wooden bar, finger displacement length^b^	4.00 (11.93) *N* = 27	8.38 (14.26) *N* = 24	7.35 (14.75) *N* = 26	16.05 (22.93) *N* = 19	9.15 (16.27) *N* = 26	11.79 (20.48) *N* = 19	3.60 (−5.27,12.46)	0.422

TRI-HFT, Toronto Rehabilitation Institute Hand Function Test; FES, functional electrical stimulation; CT, conventional therapy; CI, confidence interval; N, number of participants; SD, standard deviation.

^a^ Data reported as: mean (SD), unless otherwise specified.

^b^ For these subscale scores, a score of 0 was used when a task could not be performed and multiple imputation approach was used in the analysis.

The outcome data for the SCI-QOL basic mobility, fine motor, manual wheelchair, power wheelchair, self-care, independence, pain behavior, pain interference, and satisfaction with social roles and activities subscales are presented in Table 8. There were minimal gains in both groups on several subscales, however the standard deviations were high, and no differences were statistically significant (Table 8).

**Table 8:** SCI-QoL.

Subscale T-score^a^	Baseline		End of treatment		End of study		Outcome analysis	
	FES	CT	FES	CT	FES	CT	Effect estimate (95% CI)	*p*-value
Basic mobility	43.36 (7.69) *N* = 27	43.71 (6.74) *N* = 24	45.26 (8.80) *N* = 26	46.30 (7.85) *N* = 19	45.82 (8.22) *N* = 26	47.06 (7.69) *N* = 19	−1.27 (−4.42, 1.87)	0.425
Fine motor	40.84 (5.79) *N* = 27	42.51 (5.42) *N* = 24	43.26 (5.61) *N* = 26	45.33 (5.24) *N* = 19	43.44 (5.53) *N* = 26	44.72 (5.25) *N* = 19	−0.13 (−4.03, 3.77)	0.948
Independence	41.59 (6.30) *N* = 27	39.57 (6.00) *N* = 24	44.97 (8.43) *N* = 26	42.99 (5.17) *N* = 19	44.99 (8.02) *N* = 26	43.02 (5.34) *N* = 19	−0.71 (−3.44, 2.02)	0.612
Manual wheelchair	43.42 (6.41) *N* = 8	41.15 (8.02) *N* = 13	47.16 (10.22) *N* = 10	45.43 (7.37) *N* = 7	45.78 (6.93) *N* = 9	48.29 (4.32) *N* = 7	−0.75 (−6.95, 5.46)	0.811
Power wheelchair	43.16 (5.27) *N* = 21	45.33 (5.82) *N* = 18	45.96 (7.00) *N* = 20	48.96 (6.72) *N* = 14	45.95 (7.01) *N* = 19	49.05 (7.20) *N* = 14	−0.72 (−3.57, 2.13)	0.620
Pain behaviour	51.76 (9.15) *N* = 27	52.89 (9.28) *N* = 24	51.73 (9.05) *N* = 26	53.04 (9.63) *N* = 19	52.64 (8.35) *N* = 26	52.17 (10.19) *N* = 19	2.18 (−2.65, 7.01)	0.375
Pain interference	50.61 (8.99) *N* = 27	52.6 (9.32) *N* = 24	50.51 (8.46) *N* = 26	51.18 (8.84) *N* = 19	50.27 (7.97) *N* = 26	51.05 (9.34) *N* = 19	1.52 (−3.15, 6.19)	0.523
Self-care	40.03 (6.15) *N* = 27	42.83 (4.99) *N* = 24	43.11 (7.14) *N* = 26	44.54 (5.52) *N* = 19	43.75 (7.73) *N* = 26	45.11 (5.50) *N* = 19	0.38 (−3.40, 4.15)	0.844
Satisfaction with Social Roles and Activities	42.41 (6.46) *N* = 27	41.15 (5.16) *N* = 24	44.92 (6.00) *N* = 26	43.04 (4.00) *N* = 19	43.81 (4.67) *N* = 26	43.34 (6.23) *N* = 19	−0.26 (−2.87, 2.36)	0.847

SCI-QoL, Spinal Cord Injury Quality of Life; FES, functional electrical stimulation; CT, conventional therapy; CI, confidence interval; N, number of participants; SD, standard deviation.

^a^ Data reported as: mean (SD), unless otherwise specified.

Safety was assessed based on serious and non-serious adverse events in each group. Table 9 shows the total number of adverse events (AE) and serious adverse events (SAE) in each group and the related analysis. There were no statistically significant differences in the proportion of participants having experienced an AE or SAE between either group. The types of SAE that occurred fell into the categories of urinary, respiratory, fever/sepsis, and skin (not related to electrode sites) complications that resulted in overnight hospitalizations. One participant died while enrolled in the trial and the death was not related to study activities. One participant experienced two AE that were definitely related to the FES therapy (redness at electrode site). That same participant experienced two other AE that were possibly related to FES therapy (left dorsum hand swelling, right ventral forearm swelling).

**Table 9:** Adverse events.

Safety analysis	Descriptive	OR (95% CI)	*p*-value
Number of AE	CT: 25 FES: 41	–	–
Participants with AE	CT: 12/12 (50%) FES: 10/17 (37%)	0.588 (0.192, 0.800)	0.3523
Number of SAE	CT: 3 FES: 9	–	–
Participants with SAE	CT: 3/24 (12.5%) FES: 3/27 (11.1%)	0.875 (0.159, 4.809)	0.8779

OR, odds ratio; CI, confidence interval; AE, adverse events; FES, functional electrical stimulation; CT, conventional therapy; SAE, serious adverse events.

### Sensitivity analysis

The following sensitivity analyses were conducted to assess the robustness of results: (1) per-protocol, based on participants with complete data, (2) last observation carried forward, for imputed missing data, and (3) adjusted, based on age SCIM-SC baseline subscore. The results are in Table 10 and are consistent with the main analysis results.

**Table 10:** Sensitivity analyses on the primary outcome.

	Outcome variable	Effect estimate (95% CI)	*p*-value
Per Protocol^a^: SCIM-SC subscale score		Not applicable (no crossovers)	–
LOCF^a^: SCIM-SC subscale score	selfcare_total	−0.01 (−1.91, 1.90)	0.995
As Randomized, Multi-variable Adjusted^b^ SCIM-SC subscale score	selfcare_total	−0.73 (−3.04, 1.58)	0.536

CI, confidence interval; SCIM-SC, Spinal Cord Independence Measure Self-Care subscale; LOCF, last observation carried forward.

^a^ Estimate and *p*-value adjusted for baseline subscale score.

^b^ Estimate and *p*-value adjusted for age, SCIM-SC baseline subscale score.

### COVID interruption

Because the interruption due to the COVID-19 pandemic lockdown impacted 17 of the 51 participants, we analysed whether that interruption impacted the primary outcome (change in SCIM-SC subscale score at the end of treatment). Using multiple imputation analysis with the subgroup included of excluded, there was no significant effect of the primary outcome (Table 11).

**Table 11:** COVID subgroup analysis using multiple inputation.

Outcome variable	Subgroup definition	Subgroup-included Effect estimate (95% CI)	Subgroup-Excluded Effect estimate (95% CI)	Interaction *p*-value
SCIM-SC subscale score^a^	17 participants affected	−1.839 (−5.013,1.335)	1.839 (−1.335,5.013)	0.6272

^a^ Estimates and *p*-value adjusted for SCIM-SC baseline subscale score.

## Discussion

The key findings from this trial indicate that FES therapy (delivered using the MyndMove stimulator) and conventional therapy both produce more than a small meaningful change in SCIM self-care in individuals with traumatic C4–7 incomplete SCI in the subacute to early chronic time range post-injury. Additionally, FES therapy is safe and has a similar safety profile as conventional therapy.

The results of this study are different from a previous study (13) in which FES therapy using the Compex Motion stimulator (1 h) was compared to C.T. (1 h). That trial also involved individuals with C4–7 AIS B-D SCI, but they were within six months post-injury so all participants also received an additional hour of C.T. as part of their standard clinical care. Each group received 10 h of therapy per week for eight weeks, and in that situation, FES therapy was superior. Time post-injury could contribute to the difference in results in the current study as the FES group was a median of twenty-four months post-injury and the C.T. group was a median of eighteen months post-injury, as opposed to an average of two months post-injury in the previous study (13). It is known that the majority of natural recovery occurs within the first twelve months post-injury (20, 21). However, it is also known that some activity-based therapies as well as conventional therapies can improve upper extremity function in individuals well past twelve months post-injury (22), especially in individuals with incomplete tetraplegia. A pilot study comparing FES therapy (Compex Motion stimulator) to C.T. in persons greater than twenty-four months post-injury suggested more improvement over control (15), justifying including early chronic time points in the current trial. The dose of therapy could also be a contributing factor to the observed differences. In the current study (chronic), participants received 40 h of research therapy over fourteen weeks whereas in the previous study (sub-acute), participants received 40 h of research therapy and 40 h of clinical therapy over eight weeks (due to the inclusion of only sub-acute SCI which coincided with the timing of clinical therapy after an injury). By expanding the time window in the current study to include individuals up to ninety-six months/eight years post-injury, variability was introduced in regard to the amount of clinical therapy individuals may have had access to. It has been demonstrated that the type of therapy may be more important than the dose of therapy in regard to improving hand function in individuals with incomplete tetraplegia (23).

The results of the current trial must also be interpreted through the haze of the COVID-19 pandemic. The lockdown across Canada and the U.S. started ten months after the beginning of enrollment and lasted for varying lengths of time across the four study centers. Two sites were shut down for approximately three months, one site was shut down for approximately five months, and one site was shut down for approximately ten months. As shown in Supplementary Table S1, seventeen participants were paused mid-protocol due to the lockdown. When each site was able to resume in-person research activities, the paused participants were invited to resume the trial based on the plan described in the Methods. However, not each participant resumed immediately (some reasons included hesitancy to be in-person, complications that occurred while on pause, etc.). Hence, there was great variability between each of the seventeen participants related to the number of therapy sessions completed prior to the lockdown, the length of time on pause, the number of sessions repeated upon resuming trial activities, and the timing of outcome assessments relative to the first session. The effect of these variables on outcomes is not known. Additionally, the length of the COVID-19 pandemic impacted our ability to reach the randomization target of 60 participants based on power analysis. Enrollment was expected to be completed by December 2020 but was extended for an additional eight months, at which point enrollment had to be closed due to the funding timeline.

There are several limitations to the current trial. Based on the review of baseline data of SCIM-SC, GRASSP, TRI-HFT, and neurological level, it appears that participants in the FES group were likely a little more severely impaired than those in the C.T. group, which could have affected the trajectory of recovery or capacity for improvement between the two groups. Randomization was stratified by site but not based on baseline function. We suggest stratifying participants with incomplete SCI based on the baseline function of the primary outcome to allow for equal distribution between groups in future studies. Similarly, though the median time post-injury was not statistically different between the two groups, there appears to be a shift of about five months between the groups, with the FES group being slightly longer post-injury.

Categories of C.T. were prescribed for the trial, but the implementation was not standardized between sites as was done for FES therapy. Therefore, the active controls in this cohort may have received different categories of interventions based on the site's standard for C.T.

In conclusion, forty sessions of FES therapy delivered *via* the MyndMove stimulator are as effective as C.T. in producing meaningful functional improvements that persist after therapy is completed. Future *post hoc* analyses of this trial's dataset could include a responder analysis, right and left side analysis, or stratification based on baseline function.

## Data Availability

All data from this work will be maintained at the Biostatistics Unit at St. Joseph's Healthcare Hamilton. Data sharing and access to the trial dataset is incorporated into the Clinical Trial Agreements between the MyndTec Inc. and each of the Principal Investigators and Sites according to institutional requirements. Access to the trial dataset by individuals outside the study will be reviewed on a case-by-case basis.
